# Multiple metastatic malignant phyllodes tumor of the breast with tonsillar metastasis: a case report

**DOI:** 10.1186/s13104-017-2375-5

**Published:** 2017-01-19

**Authors:** Tomohiro Sera, Shinichiro Kashiwagi, Tsutomu Takashima, Yuka Asano, Wataru Goto, Nozomi Iimori, Satoru Noda, Naoyoshi Onoda, Masahiko Ohsawa, Kosei Hirakawa, Masaichi Ohira

**Affiliations:** 1 Department of Surgical Oncology, Osaka City University Graduate School of Medicine, 1-4-3 Asahi-machi, Abeno-ku, Osaka, Japan; 2 Department of Diagnostic Pathology, Osaka City University Graduate School of Medicine, 1-4-3 Asahi-machi, Abeno-ku, Osaka, Japan

**Keywords:** Phyllodes tumor, Tonsillar metastasis, Surgery, Breast tumor, Malignant

## Abstract

**Background:**

Tonsillar metastasis is very rare and accounts for only 0.8% of tonsillar tumors. And phyllodes tumor of the breast with tonsillar metastasis is very rare.

**Case presentation:**

A 57-year-old Japanese woman received surgery (partial mastectomy) of malignant phyllodes tumor. Seven months after initial surgery, pharyngeal pain, swelling, and a feeling of dyspnea developed, and tumor was found in the left palatine tonsil. Computed tomography for further evaluation showed a tonsillar lesion with contrast enhancement, and tonsillar metastasis was suspected. The metastatic lung tumors had not progressed. Laryngoscopic biopsy showed a tonsillar metastasis from the malignant phyllodes tumor. Despite the diagnosis of malignant phyllodes tumor with tonsillar and pulmonary metastases, the patient refused further treatment and died about 1 month later.

**Conclusions:**

A patient with a malignant phyllodes tumor of the breast and tonsillar metastasis was reported, along with a discussion of the relevant literature of this very rare pattern of metastasis.

## Background

Tonsillar metastasis is very rare and accounts for only 0.8% of tonsillar tumors [1]. Symptoms of tonsillar metastasis include pharyngeal pain, a pharyngeal globus sensation, dysphagia, and odynophagia. However, symptomatic tonsillar metastasis is rarely found before the primary tumor. In fact, tonsillar metastasis may be asymptomatic and found only as an incidentaloma. Meanwhile, phyllodes tumor of the breast (PTB) is a relatively rare disease in which the majority of tumors are benign. Only 10% or less of all PTBs are malignant [2–4].

Phyllodes tumor of the breast with tonsillar metastasis is very rare. A patient with PTB and tonsillar metastasis is reported.

## Case presentation

**Fig. 1:**
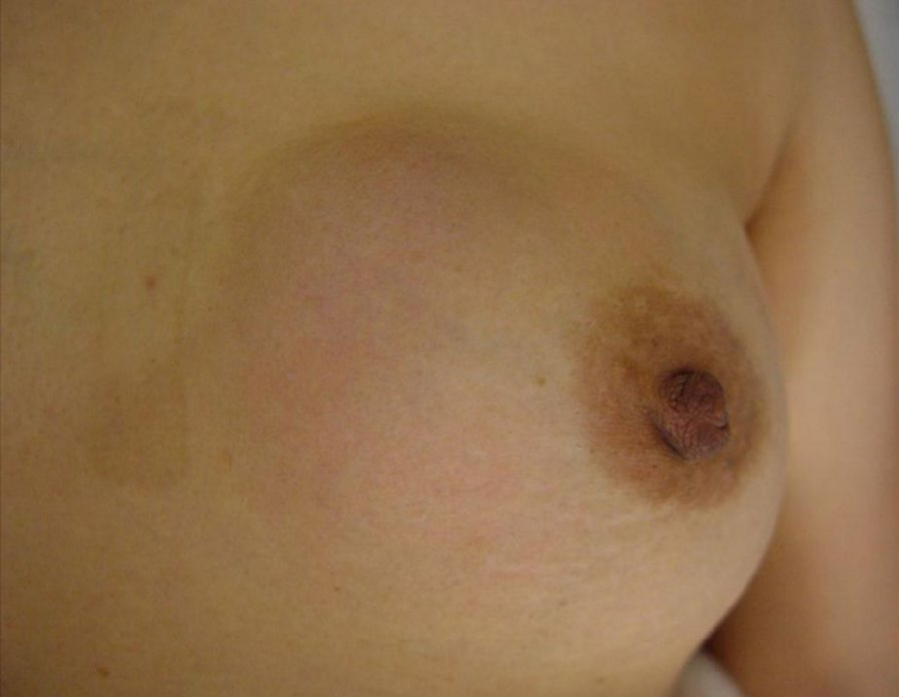
Visual palpation findings: palpation revealed a 10-cm hard, elastic, non-movable mass in the medial left breast

**Fig. 2:**
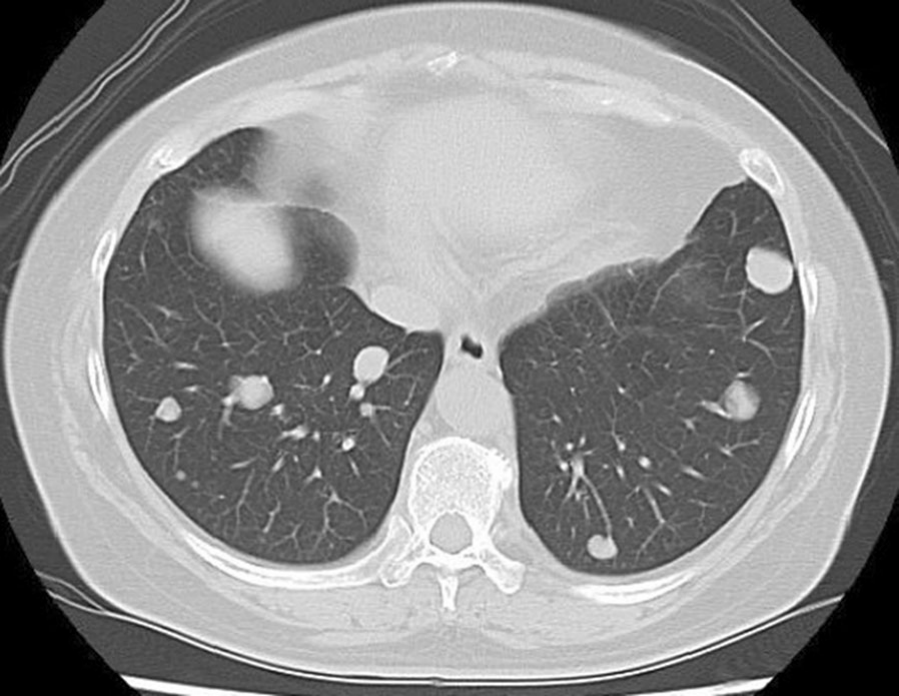
Lung CT findings: the CT images also showed multiple metastatic lung tumors

A 57-year-old Japanese woman felt a lump in her left breast more than 10 years previously that remained untreated. She now sought medical attention because the mass increased in size over the last year. Palpation revealed a 10-cm hard, non-movable mass in the medial left breast (Fig. 1). Breast ultrasonography (US) showed a large breast mass with slit formation and heterogeneous internal echoes. Fine needle aspiration cytology of the lesion suggested a phyllodes tumor (PT), but needle biopsy suggested a fibroadenoma. Malignancy could not be ruled out based on the clinical course, so vacuum-assisted breast biopsy was performed for qualitative diagnosis. A PT was suspected. Whole-body search included lung computed tomography (CT) that showed multiple metastatic lung tumors (metastatic lesion suspected), but no lymph node metastasis (Fig. 2).

A partial mastectomy with 10 mm surgical margin was performed for a definitive diagnosis, and pathological diagnosis of the resected specimen showed a malignant PTB (10.0 cm, f, ly0, v0, ew-, nuclear atypia score 3, mitotic count score 3, nuclear grade 6). Postoperative adjuvant chemotherapy was not given at the patient’s request.

**Fig. 3:**
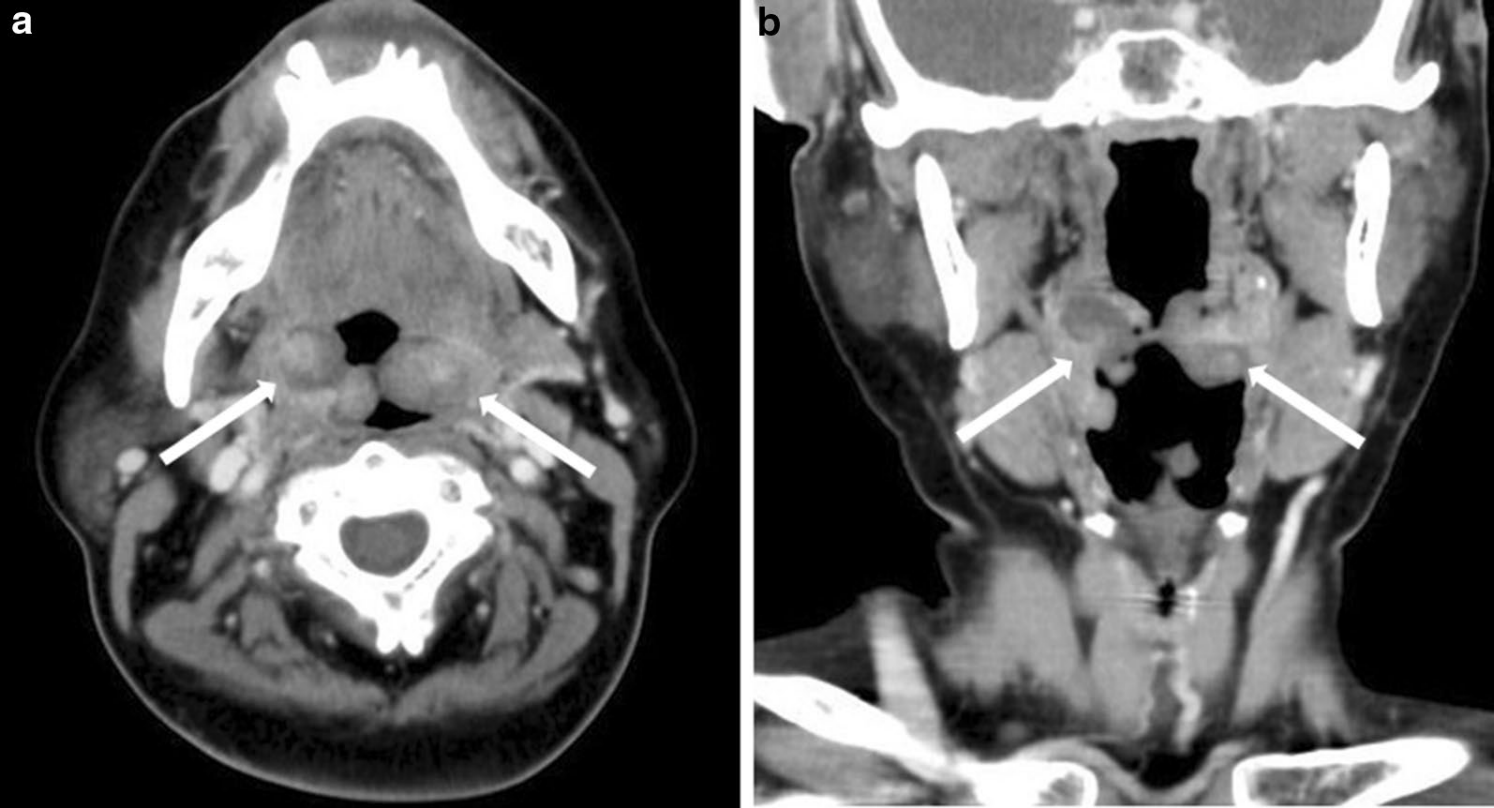
Neck CT findings: the CT images showed a tonsillar lesion with contrast enhancement, and tonsillar metastasis was suspected (**a** transverse plane, **b** coronal plane)

**Fig. 4:**
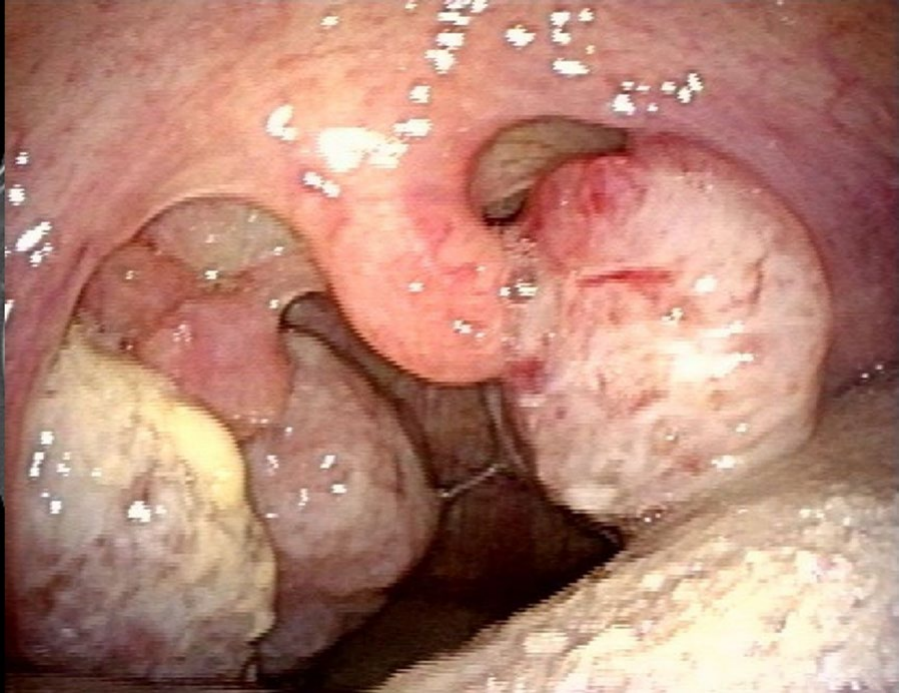
Laryngofiberscope findings. Laryngoscopic biopsy showed a tonsillar metastasis from the malignant phyllodes tumor

Two months after surgery, masses appeared at the right and left occipital, parietal, and temporal area of the head. Skin biopsy revealed skin metastases from the malignant PT, and these were resected with 10 mm surgical margin. In addition, 7 months after initial surgery, pharyngeal pain, swelling, and a feeling of dyspnea developed, and tumor was found in the left palatine tonsil. CT for further evaluation showed a tonsillar lesion with contrast enhancement, and tonsillar metastasis was suspected (Fig. 3a, b). The metastatic lung tumors had not progressed. Laryngoscopic biopsy showed a tonsillar metastasis from the malignant PT (Fig. 4). Despite the diagnosis of PTB with tonsillar and pulmonary metastases, the patient refused further treatment and died about 1 month later. Palliative care was performed according to the intention of the patient.

## Conclusions

Phyllodes tumors are histologically classified similarly to fibroadenomas as connective tissue or epithelial mixed tumors, and these account for 0.3–0.9% of all breast tumors [2]. PTs are classified as benign, borderline, and malignant based on their stromal cellularity, stromal cell atypia, stromal cell mitotic count, invasion pattern into the periphery at the tumor margins, and stromal overgrowth. About 10% of PTs are malignant [3]. Overall 5 and 10-year survival rates of 88 and 79% have been reported in PT, including 5 and 10-year survival rates of 82 and 42% for malignant PT, respectively [2]. About 25% of malignant PTs have distant metastases [2–4], most commonly to the lung, followed by the pleura, bone, and brain [5, 6]. PTB with tonsillar metastasis as seen in the present patient has only been reported once previously and had a very poor prognosis [7]. As for the past case, malignant PTB of the approximately 10 cm size was original lesion as well as this case. However, the past case received surgery (resection of the tonsillar tumor), and died of catheter infection during postoperative chemotherapy (doxorubicin and ifosfamide).

The phyllodes tumor management has traditionally consisted of surgical excision with wide tumor-free margins, generally defined by some authors as at least 10 mm [2–4]. And, adjuvant therapy has been offered to patients with malignant phyllodes tumors on an individualized basis, although its precise role is controversial. In summary, from a diagnostic and management perspective, it is important to accurately recognize malignant phyllodes tumors, which should be surgically eradicated and effectively treated at diagnosis, as these tumors have a well-established but relatively infrequent risk of metastasis and death [3].

Metastatic tonsillar tumors are rare based on the mechanism of metastases [1]. The reasons why metastatic tonsillar tumors are rare include the fact that the tonsils have no afferent lymphatic vessels and that, histologically, the tonsils are mainly reticuloendothelial cells with high ability to clear tumor. Hematogenous metastases may occur via the lungs or vertebral venous plexus (VVP). The VVP includes epidural veins and anterior vertebral veins that communicate with intercostal veins, the vena cava, the azygos venous system, and pelvic veins. This venous system has no valves, so when thoracic or abdominal pressure increases, tumor cells may spread to the VVP and metastasize in a retrograde manner to the head and neck.

Another pattern of metastasis may be direct invasion due to cervical lymph node metastasis, but lymphatic metastases are less likely because the tonsils have no afferent lymphatic vessels. The primary lesion in tonsillar metastasis is most often lung cancer, but hepatocellular carcinoma and gastric cancer have also been reported. Tonsillar metastasis associated with PTB as in the present patient is extremely rare [7].

A patient with a malignant PTB and tonsillar metastasis was reported, along with a discussion of the relevant literature of this very rare pattern of metastasis.

## Abbreviations

PTB: phyllodes tumor of the breast
US: ultrasonography
PT: phyllodes tumor
CT: computed tomography
VVP: vertebral venous plexus
